# LFHP-1c Attenuates Hepatocellular Carcinoma Viability In Vitro Independent of PGAM5

**DOI:** 10.3390/cancers17091573

**Published:** 2025-05-06

**Authors:** Ganesan Muthusamy, Chin-Chi Liu, Andrea N. Johnston

**Affiliations:** 1 School of Veterinary Medicine, Veterinary Clinical Sciences, Louisiana State University, Baton Rouge, LA 70802, USA; 2 School of Veterinary Medicine, Office of Research and Graduate Education, Louisiana State University, Baton Rouge, LA 70802, USA; cliu@lsu.edu; 3 College of Veterinary Medicine, Michigan State University, East Lansing, MI 48824, USA

**Keywords:** phosphoglycerate mutase 5, PGAM5 inhibitor, liver cancer

## Abstract

Background/Objectives: Upregulation of phosphoglycerate mutase 5 (PGAM5) is correlated with reduced survival outcomes in hepatocellular carcinoma (HCC). PGAM5 knockdown or knockout attenuates HCC growth in in vitro and in vivo models. A novel small molecule inhibitor of PGAM5, LFHP-1c, has recently been characterized. The objective of this study was to determine if LFHP-1c effectively reduces HCC viability in cell models. Methods: The hepatoma and HCC cell lines, HepG2 and HuH7, respectively, were treated with LFHP-1c. Label-free imaging was used to quantify growth. Cellular viability and reactive oxygen species (ROS) production were measured using luminescent or fluorescent assays. Expression of antioxidant and metabolic proteins was measured by immunoblot. HepG2 and HuH7 PGAM5 knockout cell lines were used as negative controls. Results: Treatment with LFHP-1c reduced cell growth and viability in HepG2 and HuH7 cell lines. Reactive oxygen species production was upregulated in both wild-type and *PGAM5* knockout cell lines following LFHP-1c exposure. Cell viability was reduced following LFHP-1c treatment in *PGAM5* knockout cell lines. Conclusions: LFHP-1c reduces hepatoma and HCC viability and enhances ROS production, but these effects are independent of PGAM5.

## Simple Summary

Hepatocellular cancer is a leading cause of cancer-related death worldwide. New targeted anti-liver cancer drugs are needed to improve survival outcomes. The phosphoglycerate mutase 5 (PGAM5) protein is upregulated in liver cancer, and its expression is correlated with reduced survival. We applied the novel PGAM5 inhibitor, LFHP-1c, to cell-based models of liver cancer, both with and without PGAM5 expression. Although a decrease in liver cancer cell viability was identified, the effect was independent of PGAM5.

## 1. Introduction

Hepatocellular carcinoma (HCC) is a leading cause of mortality worldwide, in part because of the limited number of pharmacologic options [1,2]. The identification of novel therapeutic targets is a key priority. The mitochondrial membrane-associated protein, phosphoglycerate mutase 5 (PGAM5), is upregulated in HCC and is correlated with a reduced overall patient survival [3,4,5]. Overexpression of PGAM5 has been shown to impart a pro-tumor effect through enhanced mitophagy and inhibition of apoptosis [3,6]. PGAM5 is a phosphatase that targets serine, threonine, and occasionally histidine residues [7]. Under basal conditions, PGAM5 is tethered to the mitochondrial membrane. Oxidant stress promotes cleavage of the PGAM5 N-terminus by intramitochondrial membrane proteases, freeing the catalytically active C-terminus to interact with an array of cytosolic client proteins [7,8,9,10,11].

Work by our laboratory and others has demonstrated that silencing of PGAM5 attenuates cell growth, reduces cellular ATP levels, and dysregulates lipid metabolism in cell-based and in vivo models of HCC [3,6,12,13]. A novel PGAM5 inhibitor, LFHP-1c, has recently been described and applied to models of brain injury, where it shows a protective effect [14,15,16]. LFHP-1c is a small molecule synthesized from telmisartan and 2-(1-hydroxypentyl)-benzoate that has been shown to bind PGAM5 and directly inhibit its phosphatase activity [15]. In ischemic brain injury, LFHP-1c inhibits microglial activation, attenuates endothelial cell apoptosis, and reduces neuroinflammation through 5′-adenosine monophosphate-activated protein kinase (AMPK) and nuclear factor erythroid 2-like 2 (NRF2, *NFE2L2*) activation [14,15]. LFHP-1c antagonizes the interaction of PGAM5 with the NRF2-Kelch-like ECH-associated protein 1 (KEAP1) complex. This promotes NRF2 nuclear translocation and antioxidant response element (ARE) binding, which enhances transcription of antioxidant genes such as heme oxygenase-1 (*HO-1*) and *NFE2L2* [15,17]. LFHP-1c’s impact has not been investigated in models of hepatocellular tumors. The aim of this study was to determine if LFHP-1c reduces HCC viability in cell models.

## 2. Materials and Methods

### 2.1. Cell Lines, Small Molecule Inhibitor, and Culture Conditions

The HepG2 (HB-8065) cell line was purchased from ATCC. The Huh-7D 12 (HuH7, 01042712) cell line was purchased from Millipore Sigma (Burlington, MA, USA). As previously described, the HepG2 PGAM5 knockout cell line was generated and validated by Synthego Corporation (Redwood City, CA, USA), while the Huh-7D PGAM5 knockout line was generated in our laboratory using PGAM5 CRISPR/Cas9 KO Plasmid (Santa Cruz Biotechnology, Inc., Dallas, TX, USA; sc-401300) [12]. The HepG2 cells were maintained in EMEM media (30-2003, ATCC), and HuH7 cells were maintained in DMEM, high-glucose media (25 mmol/L glucose, Gibco, Waltham, MA, USA). Both media were supplemented with 10% fetal bovine serum (VWR, Radnor, PA, USA; 45000-736) and 1% Penicillin-Streptomycin (Gibco). LFHP-1c (MedChemExpress, Monmouth Junction, NJ, USA; HY-139598) was dissolved in 10% dimethyl sulfoxide (DMSO). LFHP-1c was added to the culture media at the indicated concentrations and durations. Ten percent DMSO was used in parallel as the control treatment.

### 2.2. Cell Growth and Viability Quantification

For all assays, cells were cultured in 96-well plates at 37 °C in 5% CO_2_ for 24 h prior to treatment. Cells were treated with DMSO or LFHP-1c at the indicated concentrations and durations. Assays were repeated a minimum of 3 times independently, and each experiment had a minimum of 2 replicates. Label-free, live-cell imaging was used for cell growth quantification (Incucyte S3, Sartorius, Ann Arbor, MI, USA). ATP was quantified using the CellTiter-Glo assay (Promega, Madison, WI, USA; G7570). The assay was performed according to the manufacturer’s instructions. Luminescence was recorded with a Synergy LX multimode reader (Bio-Tek, Winooski, VT, USA). The MTT (3-(4,5-dimethylthiazol-2-yl)-2,5-diphenyltetrazolium bromide) cell viability assay (VWR, 76022-152) was performed according to the manufacturer’s instructions. The absorbance was measured at 570 nm using a Synergy LX multimode reader (Bio-Tek).

### 2.3. Quantification of Reactive Oxygen Species (ROS)

The level of intracellular ROS was quantified using the ROS Fluorometric Assay Kit (ThermoFisher, Wlatham, MA, USA; EEA019). Cells were plated at 5 × 10^5^ cells per well in 12-well plates 24 h prior to treatment. Cells were treated with DMSO or LFHP-1c at indicated concentrations for 24 h. ROS were quantified per manufacturer’s instructions. Fluorescence was measured with a multi-detection microplate reader (Synergy LX multimode reader, Bio-Tek) at 485/530 nm (excitation/emission).

### 2.4. Immunoblots

Cell lysates were prepared as previously described. (1) The protein content was measured and normalized using a BCA Protein Assay Kit (ThermoFisher, 23227). Total protein (20–40 μg) was separated by SDS-PAGE and transferred onto PVDF membranes. After blocking with 5% bovine serum albumin, the membranes were incubated with primary antibodies at 4 °C overnight, washed three times in PBS-T, and subsequently incubated with their corresponding HRP-labeled secondary antibodies. Bands were detected using ECL (Cytvia, Marlborough, MA, USA; RPN2106) on myECL Imager (ThermoFisher, G2236X). The relative intensity of proteins was analyzed using ImageJ software (National Institutes of Health, https://imagej.net/ij/, accessed on 28 April 2025). Primary antibodies included Actin (Invitrogen, Waltham, MA, USA; AB_2223496), FABP1 (Cell Signaling Technology, Danvers, MA, USA; 13368), HO1 (Invitrogen, AB_2735912), Lipin-1 (Cell Signaling Technology, 5195), NRF2 (Cell Signaling Technology, 12721), and PGAM5 (Invitrogen, AB_2900380). Secondary antibodies included goat anti-rabbit HRP (Invitrogen, AB_228341) and anti-mouse (Invitrogen, AB_228295).

### 2.5. Statistical Analyses

Statistical analyses were performed using JMP Pro 16.2.0 (SAS Institute Inc., Cary, MC, USA), and figures were generated with GraphPad Prism, version 9.4 (GraphPad Prism Software, San Diego, CA, USA). Data are expressed as mean ± standard deviation (SD) or standard error of the mean (SEM). Protein expression, ATP, MTT, and ROS were compared using ANOVA models within cell type, with LFHP-1c concentrations as the fixed effect; where appropriate, a post hoc Tukey test was used for comparisons between groups. Similar models with time as the fixed effect were used for protein expression and cell growth within LFHP-1c concentrations. The normality of residuals from the models was assessed and confirmed via Shapiro–Wilk tests. Significance was set at *p* < 0.05.

## 3. Results

### 3.1. LFHP-1c Attenuates Cell Growth and Viability in Hepatoma and Hepatocellular Carcinoma Cell Models

**Figure 1:**
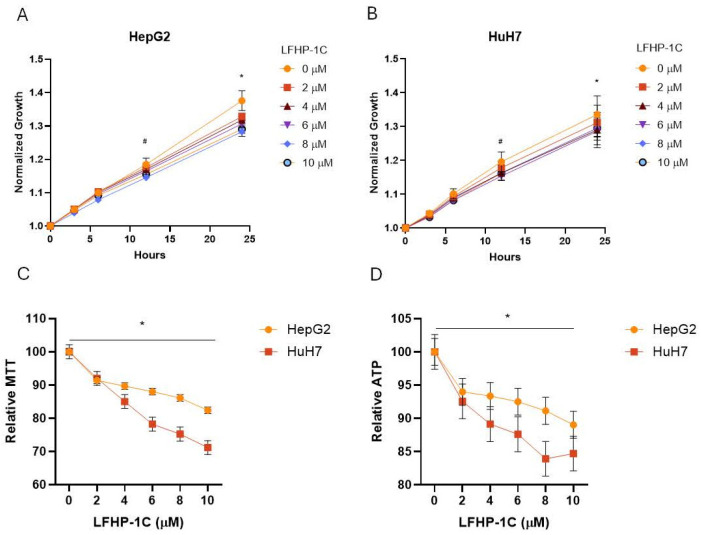
LFHP-1c decreases hepatoma and hepatocellular carcinoma cell viability. (**A**) HepG2 cells were treated with LFHP-1c (0, 2, 4, 6, 8, or 10 µM) at 0 h (h). Bright field cell area was quantified at 0, 3, 6, 12, and 24 h. Values were normalized to the 0 h measurement. The mean values and standard deviations (SD; n = 3 experiments, 2 replicates per experiment) are indicated; pairwise comparison to 0 µM: # *p* < 0.001 at 8 and 10 µM, * *p* < 0.001 at 2–10 µM. (**B**) HuH7 cells were treated with LFHP-1c (0, 2, 4, 6, 8, or 10 µM) at 0 h. Bright field cell area was quantified at 0, 3, 6, 12, and 24 h. Values were normalized to the 0 h measurement. The mean values and standard deviations (SD; n = 3 experiments, 2 replicates per experiment) are indicated; pairwise comparison to 0 µM: # *p* < 0.001 at 4–10 µM, * *p* < 0.001 at 2–10 µM. (**C**) HepG2 and HuH7 cells were treated with LFHP-1c (0, 2, 4, 6, 8, or 10 µM) at 0 h. MTT concentration was measured at 24 h. Values were normalized to the 0 µM measurement. The mean values and SD (n = 3 experiments, 3 replicates per experiment) are indicated; pairwise comparison to 0 µM: * *p* < 0.001 at 2–10 µM. (**D**) HepG2 and HuH7 cells were treated with LFHP-1c (0, 2, 4, 6, 8, or 10 µM) at 0 h. ATP concentration was measured at 24 h. Values were normalized to the 0 µM measurement. The mean values and SDs (n = 3 experiments, 3 replicates per experiment) are displayed; pairwise comparison to 0 µM: * *p* < 0.001 at 2–10 µM.

Treatment of the hepatoma and hepatocellular carcinoma cell lines, HepG2 and HuH7, respectively, with the novel PGAM5 inhibitor LFHP-1c inhibited cell growth after 24 h at all concentrations tested (Figure 1A,B). Assessment of cell viability was quantified by MTT reduction and ATP levels. Twenty-four-hour exposure to LFHP-1c significantly attenuated viability in both cell lines at all concentrations tested compared to DMSO-treated control cells (Figure 1C,D).

### 3.2. LFHP-1c Does Not Increase NRF2 Expression

**Figure 2:**
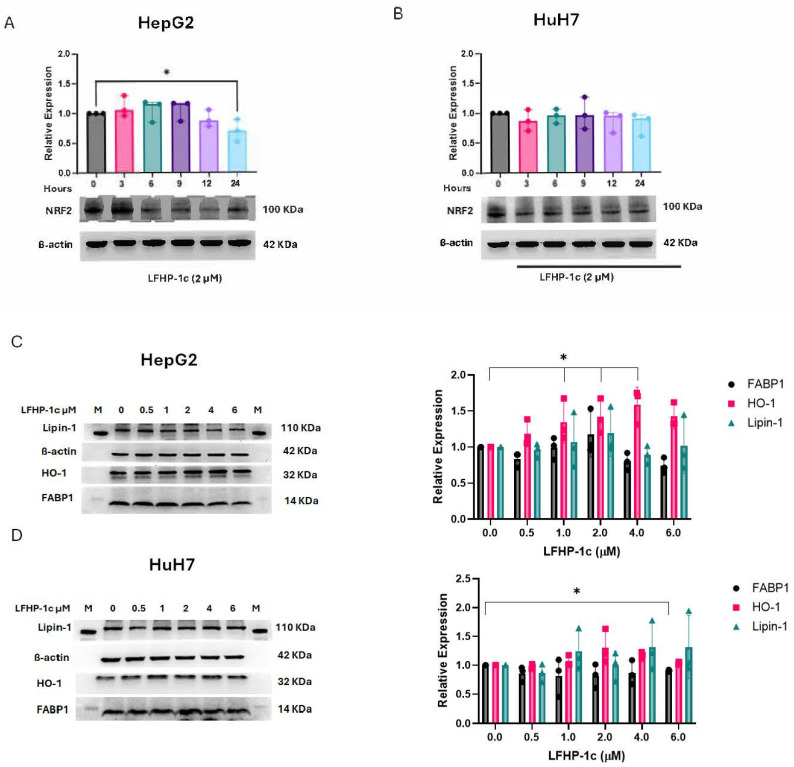
Effect of LFHP-1c on antioxidant and metabolic protein expression. (**A**) NRF2 protein expression in HepG2 cells treated with DMSO (0 µM) or LFHP-1c (2 µM) for 3, 6, 9, 12, or 24 h was determined by immunoblot (n = 3 experiments). A representative immunoblot of NRF2 and ß-actin is displayed. Protein expression was quantified by densitometry (ImageJ). Mean relative values ± SDs normalized to ß-actin is displayed for NRF2. (**B**) NRF2 protein expression in HuH7 cells treated with DMSO (0 µM) or LFHP-1c (2 µM) for 3, 6, 9, 12, or 24 h was determined by immunoblot (n = 3 experiments). A representative immunoblot of NRF2 and ß-actin is displayed. Protein expression was quantified by densitometry (ImageJ). Mean relative values ± SDs normalized to ß-actin are displayed for NRF2. (**C**) Lipin-1, HO-1, and FABP-1 protein expression in HepG2 cells treated with DMSO (0 µM) or LFHP-1c (0.5, 1, 2, 4, 6 µM) for 24 h was determined by immunoblot (n = 3 experiments). Representative immunoblots of NRF2 and ß-actin is displayed. Protein expression was quantified by densitometry (ImageJ). Mean relative values ± SDs normalized to ß-actin are displayed for Lipin-1, HO-1, and FABP-1. (**D**) NRF2 protein expression in HuH7 cells treated with DMSO or LFHP-1c (0.5, 1, 2, 4, 6 µM) for 24 h was determined by immunoblot (n = 3 experiments). Representative immunoblots of Lipin-1, HO-1, and FABP-1, and ß-actin are displayed. (**D**) Protein expression was quantified by densitometry (ImageJ). Mean relative values ± SDs normalized to ß-actin are displayed for Lipin-1, HO-1, and FABP-1. * *p* < 0.05. The uncropped bolts are shown in Supplementary Materials.

The silencing of PGAM5 in HepG2 has been shown to reduce antioxidant gene expression, including NRF2 and HO-1 [18]. In models of brain ischemia and traumatic brain injury, upregulation of NRF2 expression was used as a surrogate marker of PGAM5 inhibition following treatment with LFHP-1c. In HepG2 cells, NRF2 expression significantly decreased after a 24 h exposure to LFHP-1c at 2 µM (Figure 2A). There was no significant difference in NRF2 expression in HuH7 at any time point following LFHP-1c treatment (Figure 2B). To further assess the impact of LFHP-1c treatment, expression of the antioxidant protein marker HO-1 was measured via immunoblot. In HepG2 cells, HO-1 expression was increased after 24 h of LFHP-1c treatment at 1, 2, and 4 µM, but HO-1 expression did not increase in HuH7 cells. The deletion of PGAM5 modifies lipid metabolism in HCC. Because lipin-1 is a PGAM5 substrate, knockout of PGAM5 specifically downregulates fatty acid binding protein-1 (FABP1) expression [12,13,19]. Therefore, the expression of lipin-1 and FABP-1 was quantified by immunoblot following LFHP-1c exposure. FABP1 protein expression was significantly downregulated at 24 h when HuH7 cells were treated with 6 µM of LFHP-1c but was not reduced in HepG2 cells at any concentration tested (Figure 2C,D). The expression of lipin-1, a PGAM5 substrate, was unchanged after treatment with LFHP-1c in HepG2 and HuH7 cells [13].

### 3.3. LFHP-1c Reduces Cell Viability and Promotes ROS Generation Independent of PGAM5

**Figure 3:**
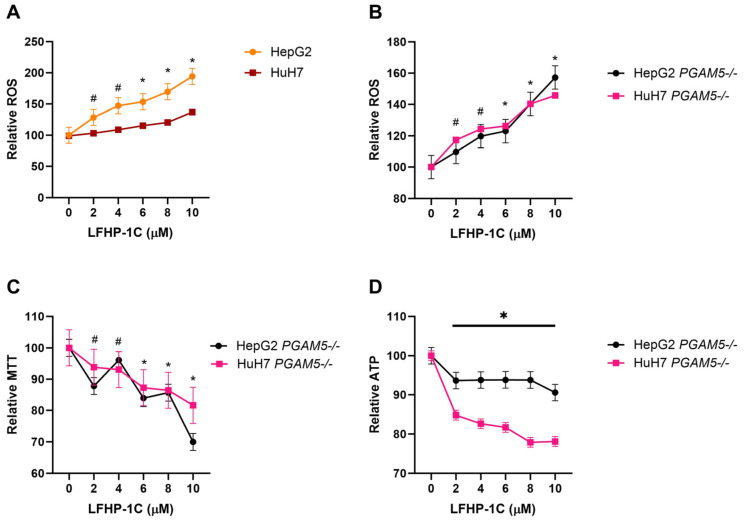
LFHP-1c elevates ROS levels and decreases cell viability independent of PGAM5 expression. (**A**) HepG2 and HuH7 cells were treated with LFHP-1c (0, 2, 4, 6, 8, or 10 µM) at 0 h. ROS was quantified at 24 h. Values were normalized to the 0 h measurement. The mean values and standard deviations (SD; n = 3 experiments, 3 replicates per experiment) are displayed; pairwise comparison to 0 µM: # *p* < 0.001 HepG2 at 2 and 4 µM, * *p* < 0.001 HepG2 and HuH7 at 6–10 µM. (**B**) HepG2 and HuH7 PGAM5 knockout cells were treated with LFHP-1c (0, 2, 4, 6, 8, or 10 µM) at 0 h. ROS was quantified at 24 h. Values were normalized to the 0 h measurement. The mean values and standard deviations (SD; n = 3 experiments, 2 replicates per experiment) are displayed; pairwise comparison to 0 µM: # *p* < 0.001 HuH7 at 2 and 4 µM, * *p* < 0.001 HepG2 and HuH7 at 6–10 µM. (**C**) HepG2 and HuH7 PGAM5 knockout cells were treated with LFHP-1c (0, 2, 4, 6, 8, or 10 µM) at 0 h. MTT concentration was measured at 24 h. Values were normalized to the 0 µM measurement. The mean values and SD (n = 3 experiments, 3 replicates per experiment) are indicated; pairwise comparison to 0 µM: # *p* < 0.001 HepG2 at 2 and 4 µM * *p* < 0.001 HepG2 and HuH7 at 6–10 µM. M-protein marker. (**D**) HepG2 and HuH7 PGAM5 knockout cells were treated with LFHP-1c (0, 2, 4, 6, 8, or 10 µM) at 0 h. ATP concentration was measured at 24 h. Values were normalized to the 0 µM measurement. The mean values and SDs (n = 3 experiments, 3 replicates per experiment) are displayed for pairwise comparison to 0 µM: * *p* < 0.001 for HepG2 and HuH7 at 2–10 µM. M-protein marker.

Reactive oxygen species production was upregulated in HepG2 (LFHP-1c [2–10 µM]) and HuH7 (LFHP-1c [6–10 µM]) cells after 24 h of LFHP-1c exposure (Figure 3A). Surprisingly, these results were recapitulated in HepG2 and HuH7 PGAM5 knockout cells (Figure 3B). Treatment with LFHP-1c also significantly reduced viability in PGAM5 knockout cells, indicating that the anti-viability effects of the small molecule inhibitor are independent of PGAM5 binding and phosphatase inhibition (Figure 3C,D).

## 4. Discussion

Previous investigations demonstrated that LFHP-1c binds to PGAM5 and inhibits its protein phosphatase activity in vitro [15]. The small molecule inhibitor has shown promise as a therapeutic in models of stroke and traumatic brain injury [16]. Inhibition of PGAM5 by LFHP-1c prevented NRF2 cytosolic tethering in the ternary NRF2-KEAP1-PGAM5 complex, promoting transcription of antioxidant genes. We hypothesize that the difference in NRF2 and HO-1 expression in HepG2 compared to HuH7 cells is explained by the greater relative increase in relative ROS production in HepG2 cells. NRF2 translocation to the nucleus promotes HO-1 expression, which negatively feeds back to reduce NRF2 expression [18]. PGAM5 may also have divergent roles in hepatocellular oxidant injury. In murine models of metabolic disease-associated steatohepatitis, PGAM5 knockout enhanced or inhibited oxidant injury depending on dietary context [19,20]. In cancer, overexpression of PGAM5 promotes antioxidant tumor defense mechanisms [3,6]. Pharmacological antagonism of PGAM5 may have therapeutic relevance in HCC, as demonstrated in model cell lines [21].

## 5. Conclusions

The results reported herein demonstrate that LFHP-1c reduces the viability and increases ROS production in commonly utilized hepatoma and HCC cell lines; however, these findings were recapitulated in PGAM5 knockout HuH7 and HepG2 cells. The latter discovery suggests that LFHP-1c has off-target effects limiting its relevance in mechanistic studies investigating PGAM5’s role in hepatocellular carcinoma. The findings that NRF2 expression is reduced in HepG2 cells, FABP1 expression is attenuated in HuH7 cells, and ROS production is increased in both cell lines are expected and align with PGAM5 inhibition by LFHP-1c. Although the mechanism by which LFHP-1c works in HCC is in question, the small molecule’s impact on cell viability may still be worth interrogating in cancer models. While the work described in this brief research report is limited in scope, the primary conclusion is relevant to future studies: LFHP-1c inhibits the viability of HepG2 and HuH7 cells independent of PGAM5.

## Data Availability

The datasets generated for this study can be found in Figshare, DOI https://figshare.com/articles/dataset/Johnston_LFHP1c_short_report_data/27098377. Access date 24 September 2024.

## Supplementary Materials

The following supporting information can be downloaded at: https://www.mdpi.com/article/10.3390/cancers17091573/s1, The uncropped bolts.

## Abbreviations

The following abbreviations are used in this manuscript:

AMPK	5′-adenosine monophosphate-activated protein kinase
DMSO	Dimethyl sulfoxide
KEAP	Kelch-like ECH-associated protein 1
MTT	3-(4,5-dimethylthiazol-2-yl)-2,5-diphenyltetrazolium bromide
NRF2/*NFE2L2*	Nuclear factor erythroid 2-like 2
PAGE	Polyacrylamide gel electrophoresis
PGAM5	Phosphoglycerate mutase 5
ROS	Reactive oxygen species
SDS	Sodium dodecyl sulfate
