# Supplementary material for: LFHP-1c Attenuates Hepatocellular Carcinoma Viability In Vitro Independent of PGAM5
Source: Cancers (Basel). 2025 May 6;17(9):1573. doi: 10.3390/cancers17091573 (PMC12071907; doi:10.3390/cancers17091573)
Supplement: Supplementary file 1 [file cancers-17-01573-s001.zip › cancers-3556057-supplementary.pdf]

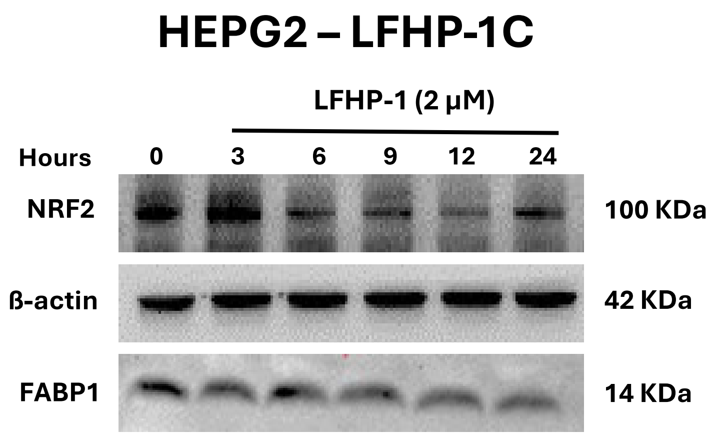
HEPG2 – LFHP-1C
LFHP-1 (2 μM)
Hours 0 3 6 9 12 24
NRF2
100 KDa
ß-actin
42 KDa
FABP1
14 KDa

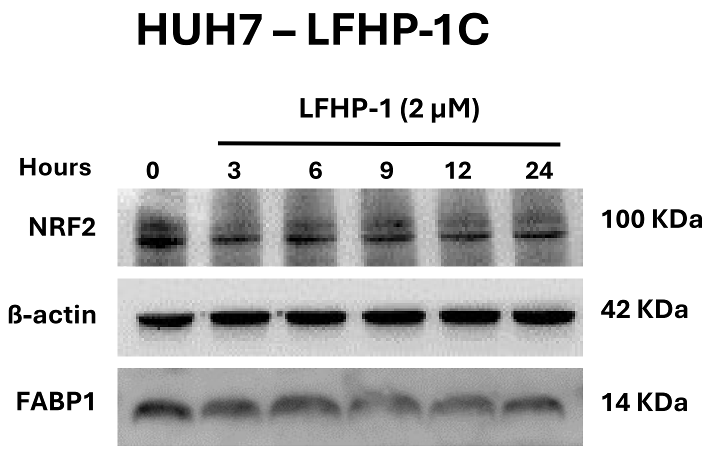
HUH7 – LFHP-1C
LFHP-1 (2 μM)
Hours 0 3 6 9 12 24
NRF2
100 KDa
ß-actin
42 KDa
FABP1
14 KDa

HEPG2 – LFHP-1C

Membrane 1

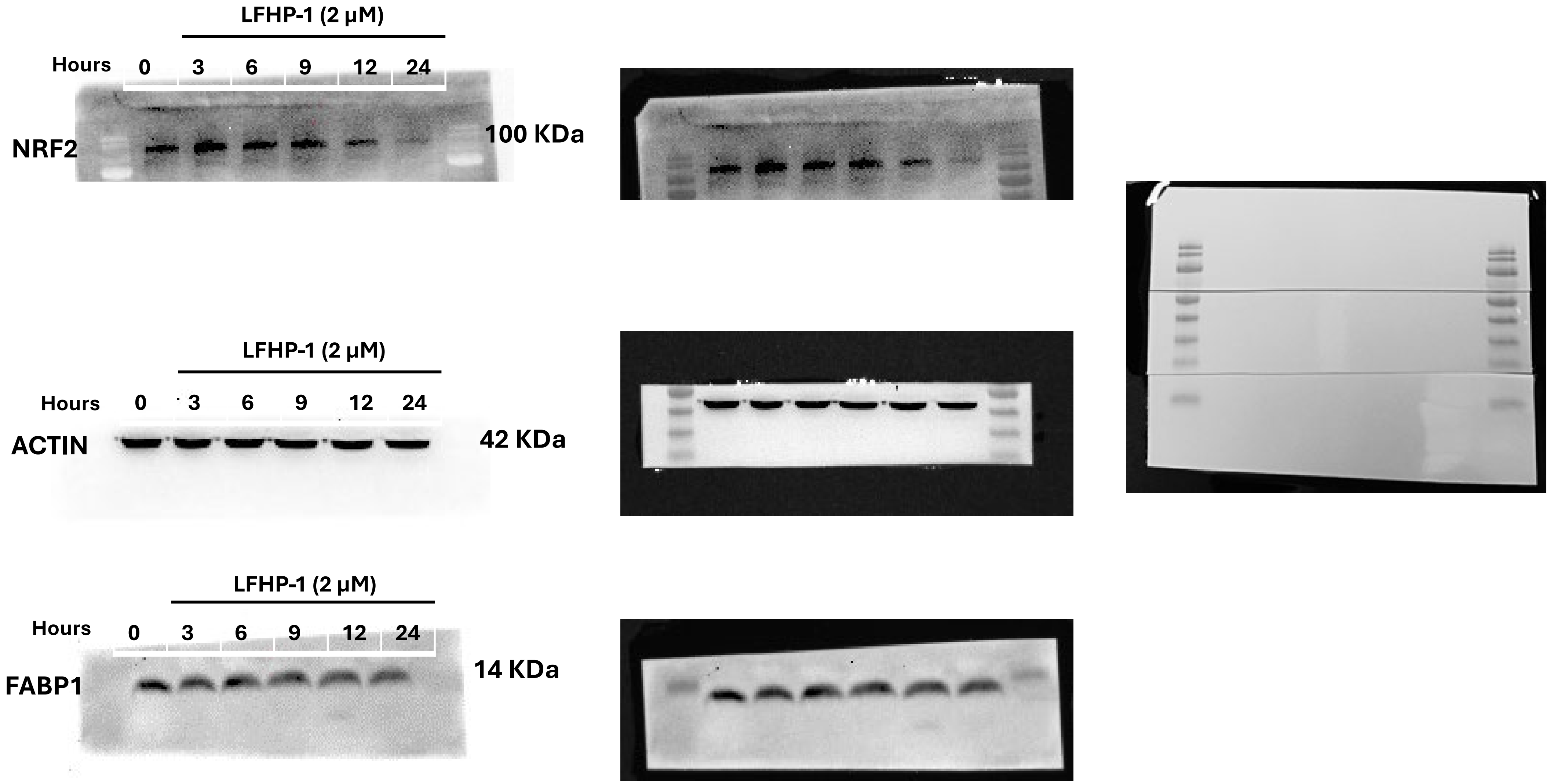

Membrane 2

HEPG2 – LFHP-1C (BSA BLOCKING)

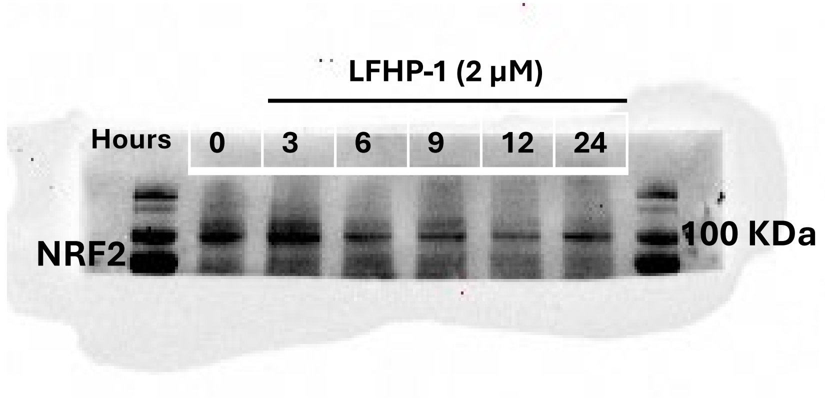

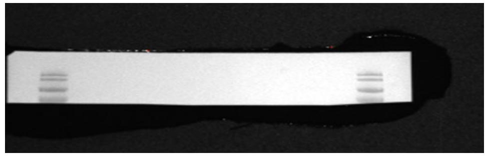

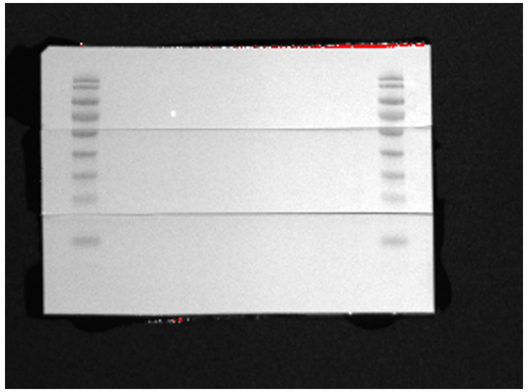

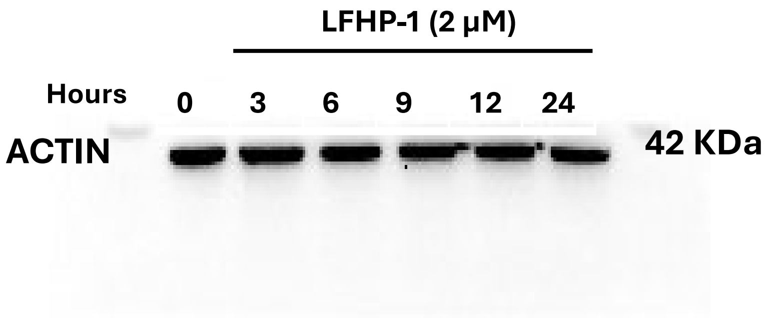

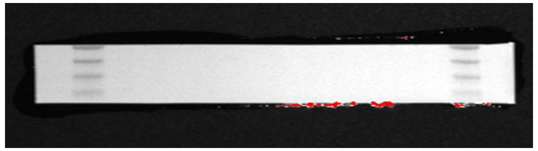

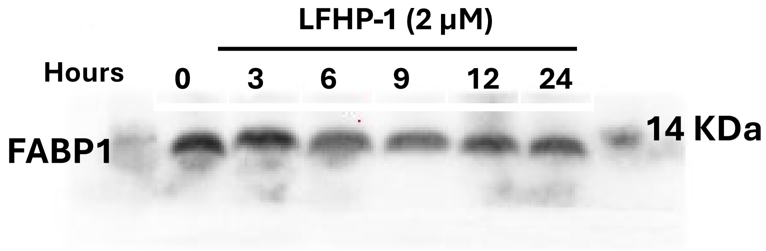

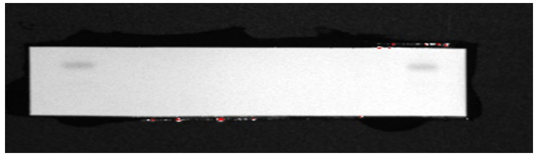

Membrane 3

HEPG2_LFHP-1c (2 μM)_0-24H

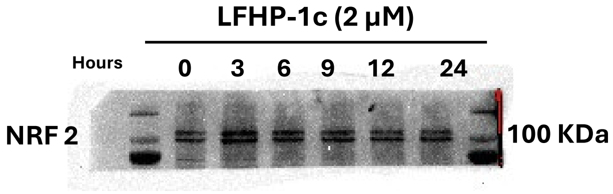

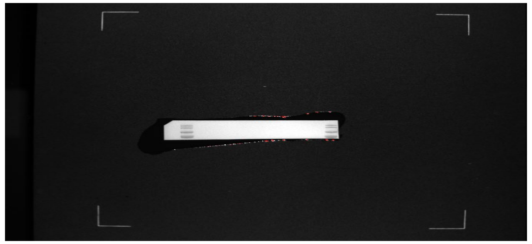

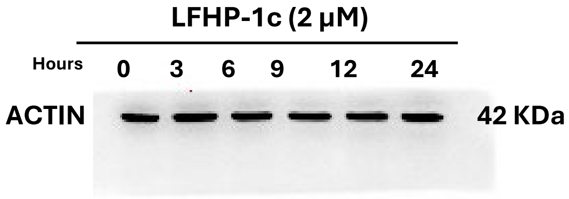

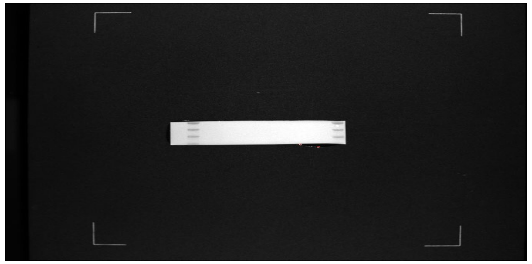

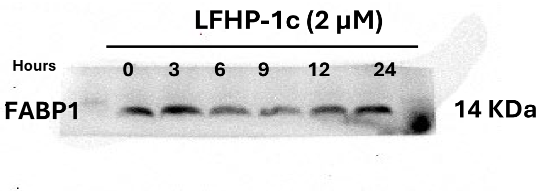

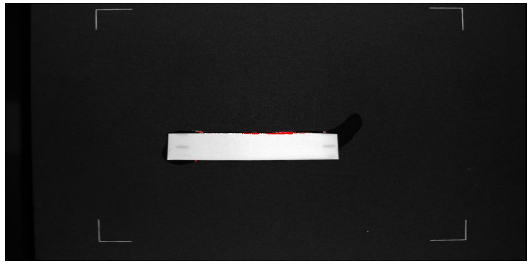

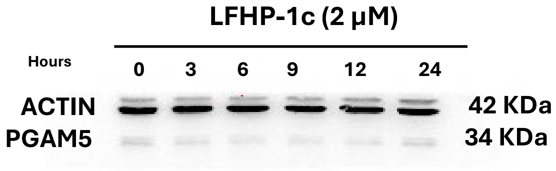

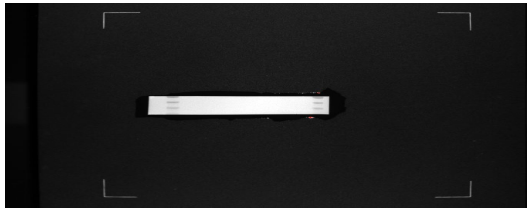

Membrane 1

HUH7 – LFHP-1C

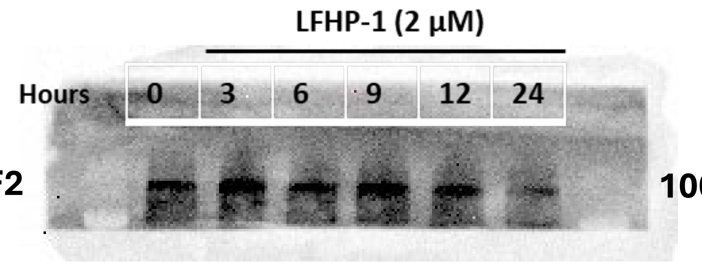

NRF2

100 KDa

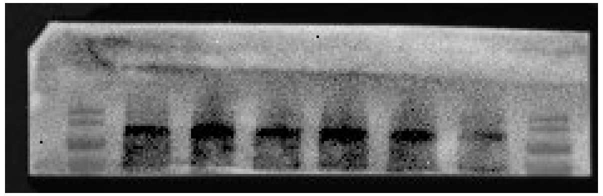

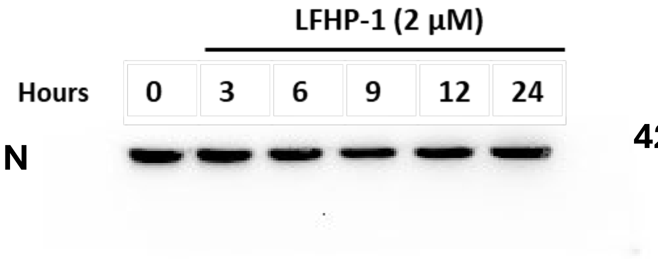

ACTIN

42 KDa

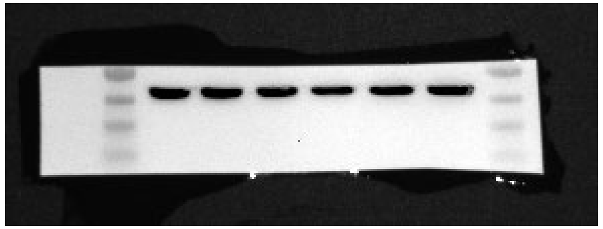

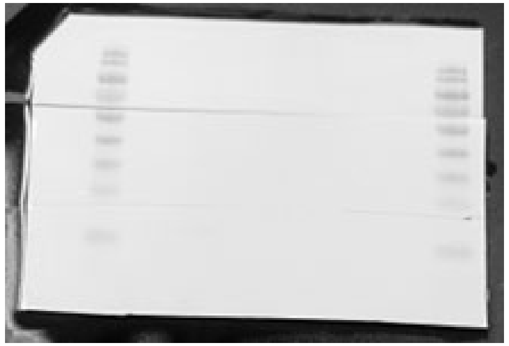

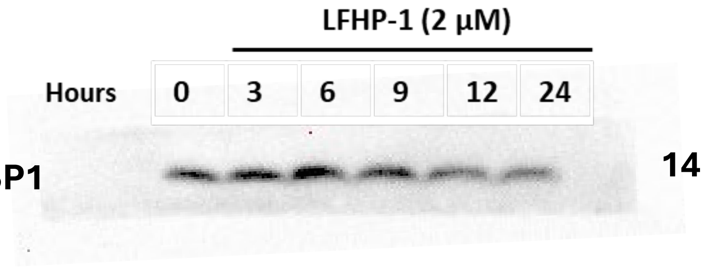

FABP1

14 KDa

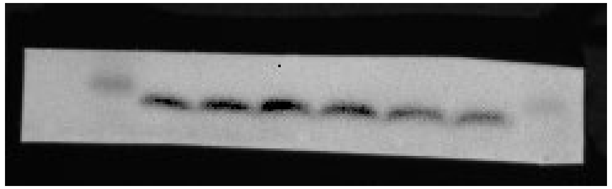

Membrane 2

HUH7 – LFHP-1C (BSA BLOCKING)

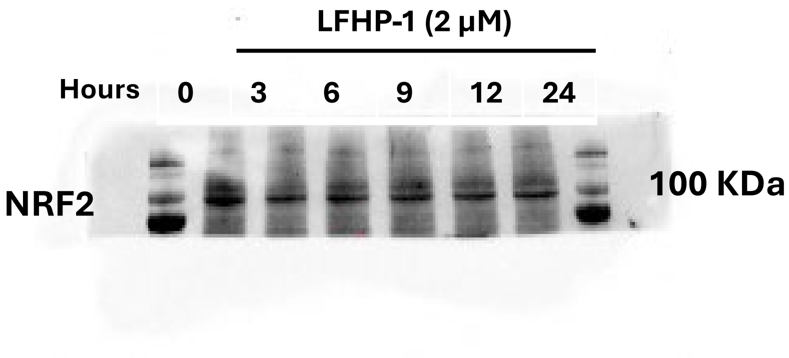

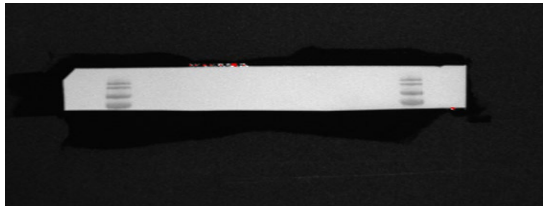

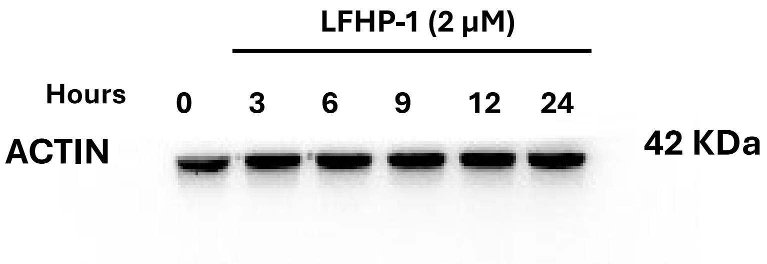

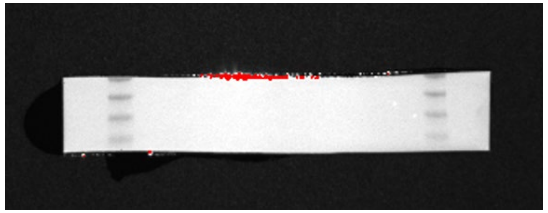

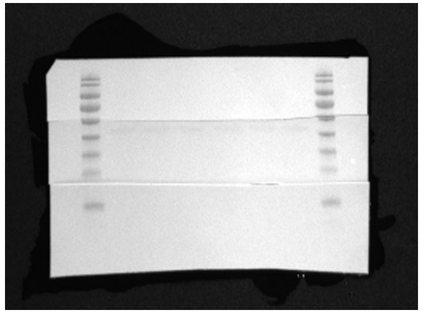

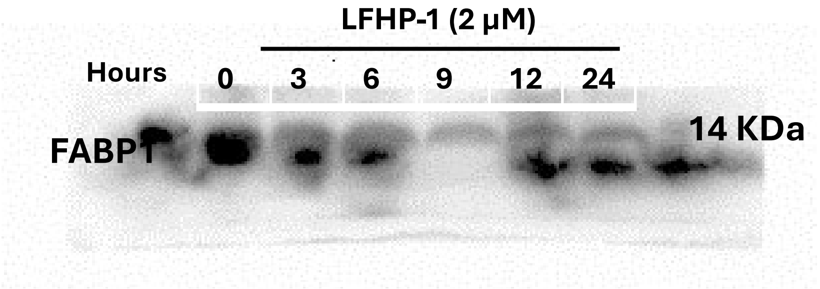

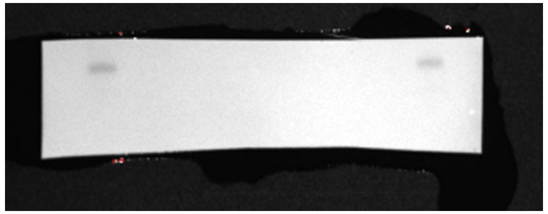

HUH7_LFHP-1c (2 μM)_0-24H

Membrane 3

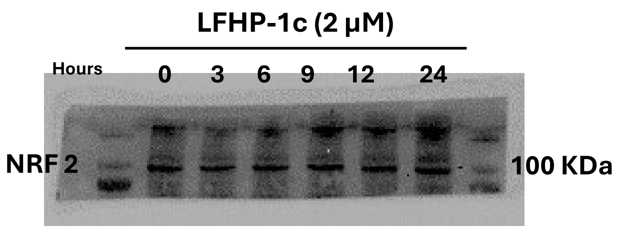

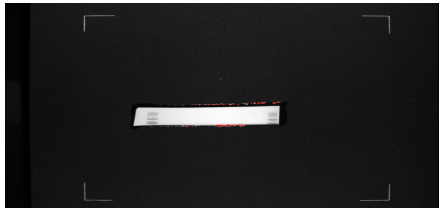

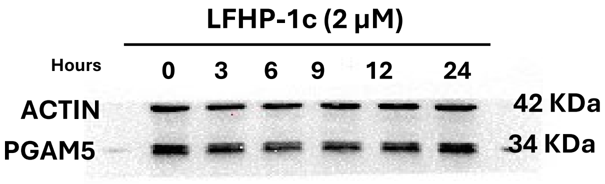

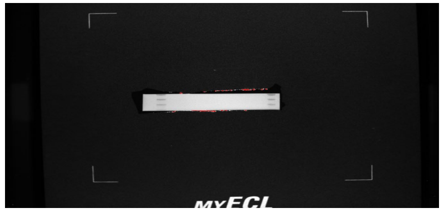

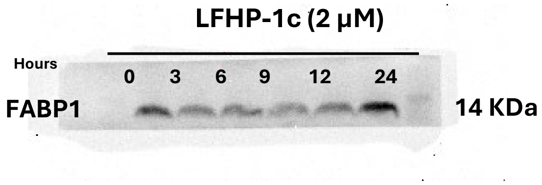

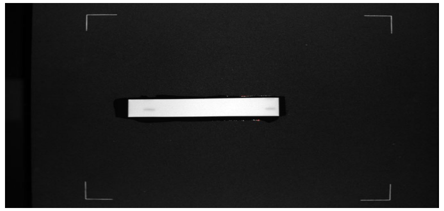

Immunoblot Densitometry Ratios

Fig 2A

**HepG2**	Time (hours)	0			3			6			9			12			24		
		Exp 1	Exp 2	Exp 3	Exp 1	Exp 2	Exp 3	Exp 1	Exp 2	Exp 3	Exp 1	Exp 2	Exp 3	Exp 1	Exp 2	Exp 3	Exp 1	Exp 2	Exp 3
	Densitometry ratio	1	1	1	1.05024	0.960641	1.299042	1.151921	0.846402	1.184833	1.161198	0.863304	1.165446	0.777377	0.867691	1.054951	0.516181	0.701043	0.890304
**Huh7**	Time (hours)	0			3			6			9			12			24		
		Exp 1	Exp 2	Exp 3	Exp 1	Exp 2	Exp 3	Exp 1	Exp 2	Exp 3	Exp 1	Exp 2	Exp 3	Exp 1	Exp 2	Exp 3	Exp 1	Exp 2	Exp 3
	Densitometry ratio	1	1	1	1.057506	0.703286	0.864101	0.958046	0.823521	1.064423	1.262202	0.7291	0.955428	0.947804	0.659805	0.998399	0.600461	0.899544	0.958984

Fig 2B

		Densitometry ratio per antibody								
**HepG2**	*LFHP-1C*	FABP1			HO-1			Lipin-1		
	Concentration	Exp 1	Exp 2	Exp 3	Exp 1	Exp 2	Exp 3	Exp 1	Exp 2	Exp 3
	0	1	1	1	1	1	1	1	1	1
	0.5	0.894561	0.715146	0.893441	1.383348	1.052421	1.107705	0.85622	1.039175	0.98592
	1	1.123739	0.823341	1.021437	1.673716	1.129583	1.23748	0.734907	1.48444	0.989098
	2	1.534529	1.032517	0.956787	1.674971	1.377665	1.196686	0.980573	1.566263	1.031129
	4	0.919444	0.680751	0.797505	1.75149	1.311253	1.70626	0.893336	1.015865	0.766797
	6	0.855152	0.713431	0.64167	1.328657	1.320939	1.621382	0.891952	1.449725	0.714542

		Densitometry ratio per antibody								
**Huh7**	*LFHP-1C*	FABP1			HO-1			Lipin-1		
	Concentration	Exp 1	Exp 2	Exp 3	Exp 1	Exp 2	Exp 3	Exp 1	Exp 2	Exp 3
	0	1	1	1	1	1	1	1	1	1
	0.5	0.901559	0.709718	0.95788	1.019036	0.975873	0.93562	0.707815	1.017208	0.87063
	1	0.905758	0.450015	1.092767	1.004013	1.173573	1.022116	1.125431	1.641806	0.958955
	2	0.837172	0.604912	1.014094	1.064621	1.632204	1.203145	1.047889	1.209991	0.720709
	4	0.829637	0.671753	1.08898	1.115868	1.178753	1.227144	1.21047	1.777609	0.944091
	6	0.921737	0.904686	0.886658	1.048816	1.012384	1.059257	1.047122	1.94337	0.950283

HEPG2_LFHP-1_2 uM_0-24 H

EXPERIMENT 1

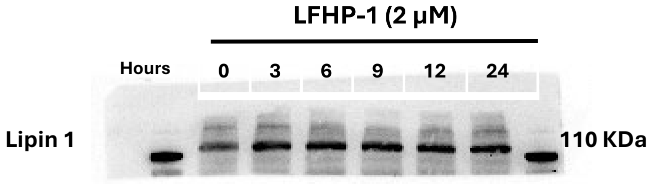

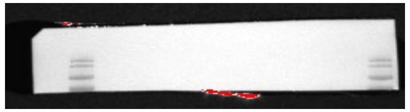

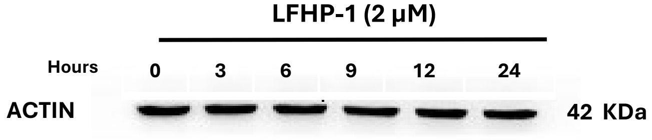

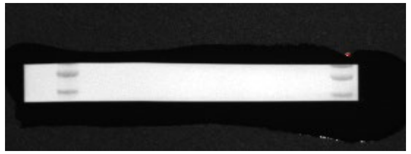

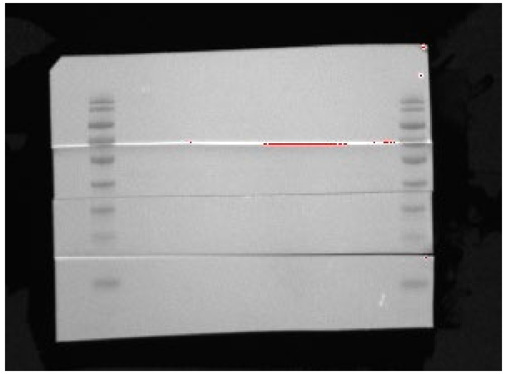

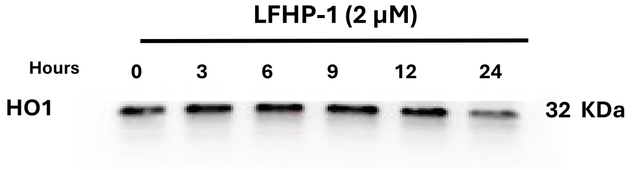

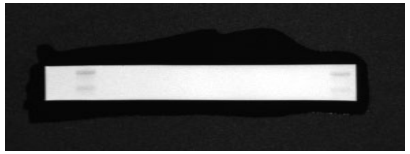

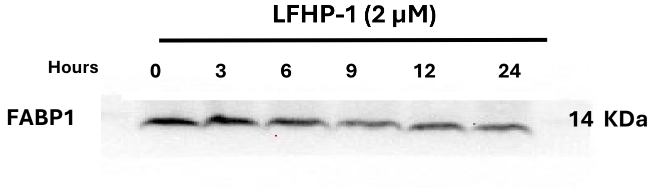

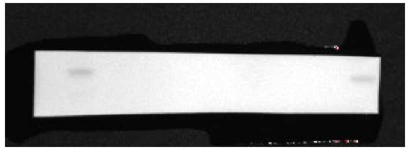

HEPG2_LFHP-1_0.5-6 uM

EXPERIMENT 1

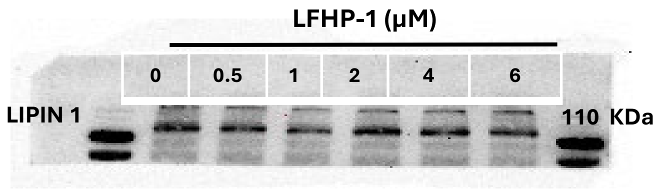

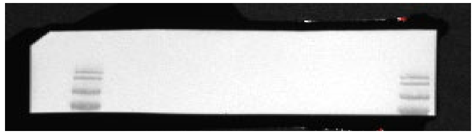

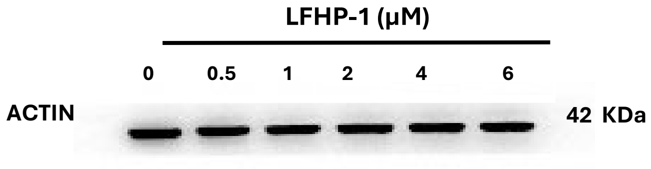

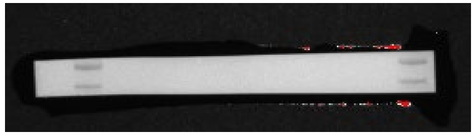

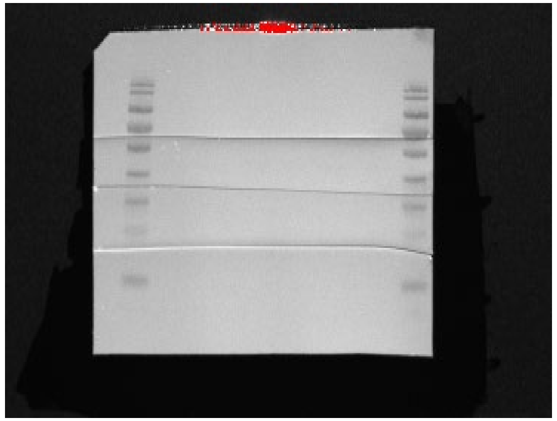

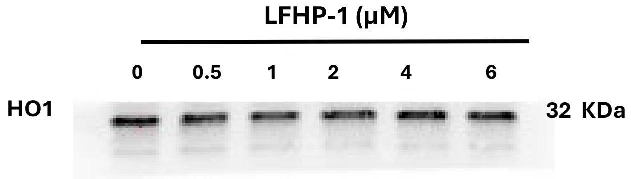

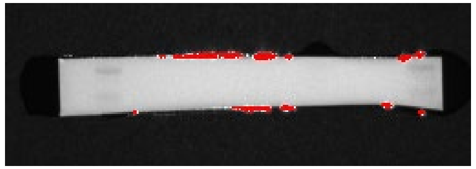

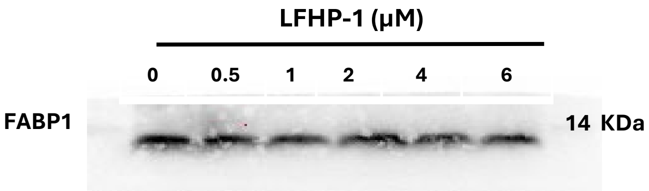

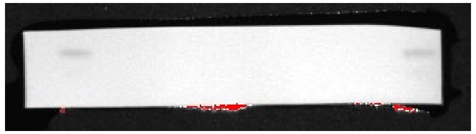

HUH7_ LFHP-1_0.5-6 uM

EXPERIMENT 1

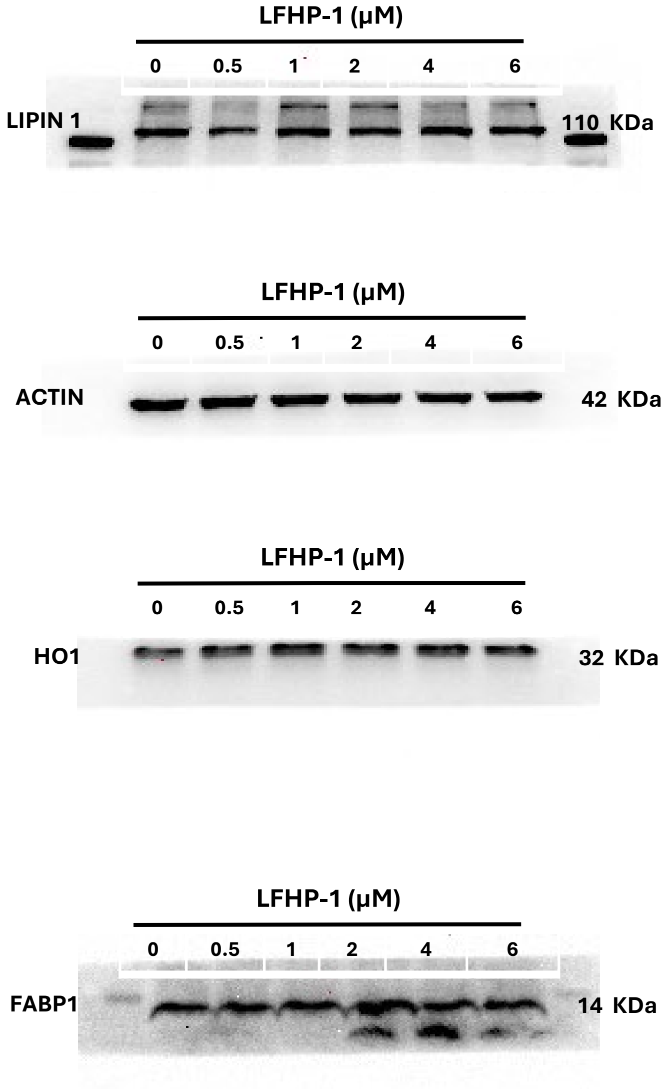

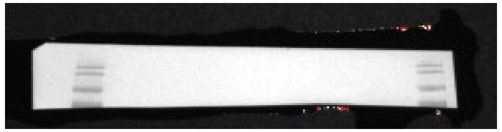

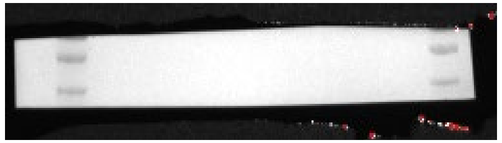

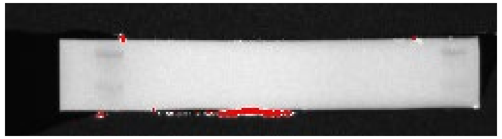

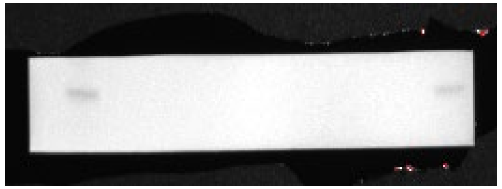

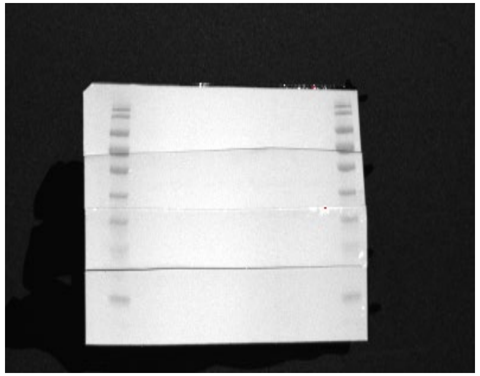

HUH7_LFHP-1_2 uM_0-24 H

EXPERIMENT 1

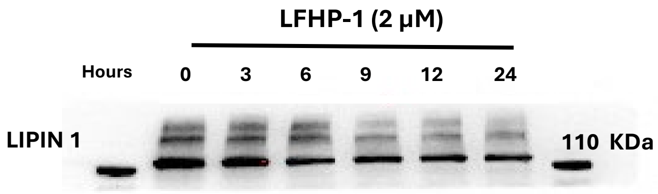

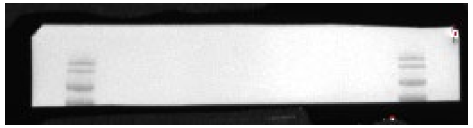

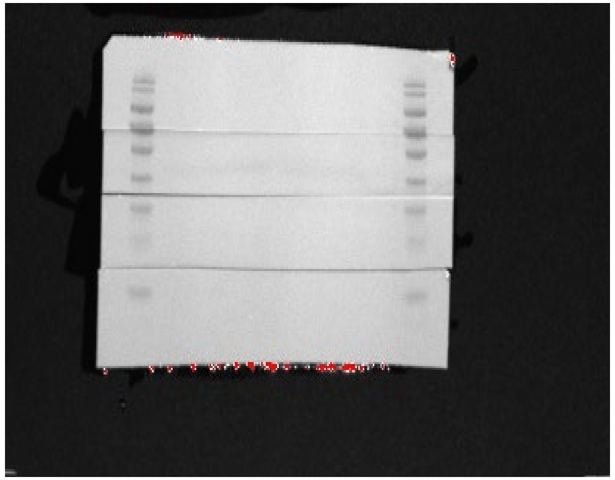

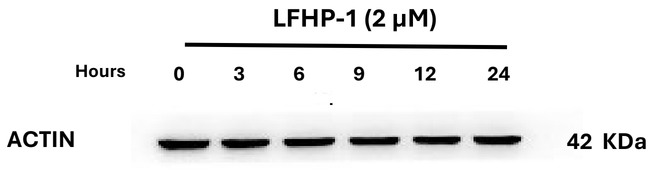

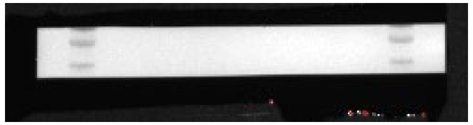

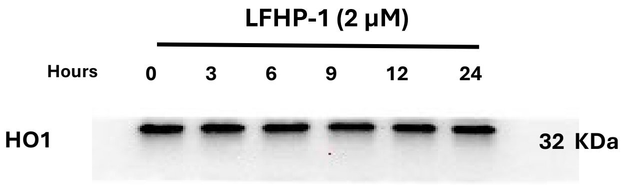

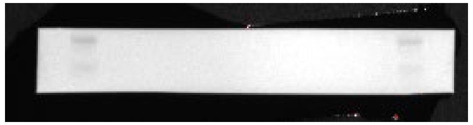

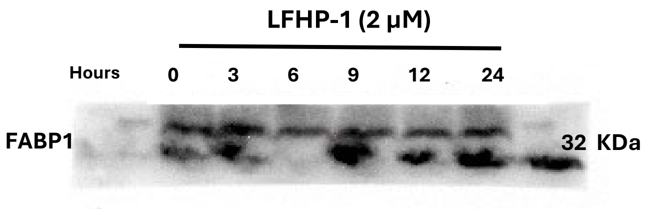

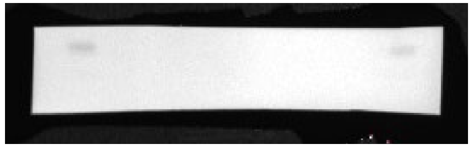

HEPG2_LFHP-1_0.5-6 uM

EXPERIMENT 2

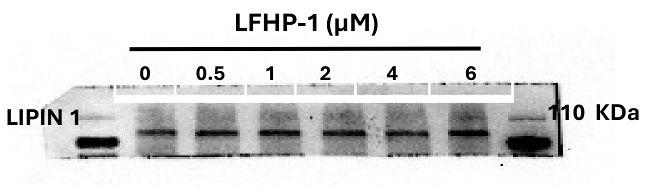

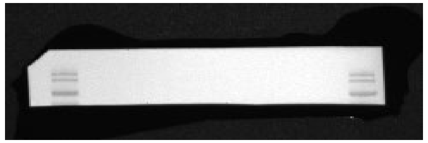

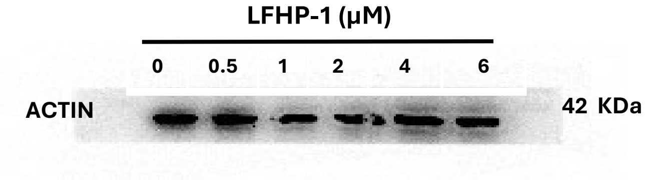

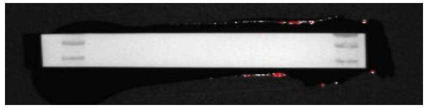

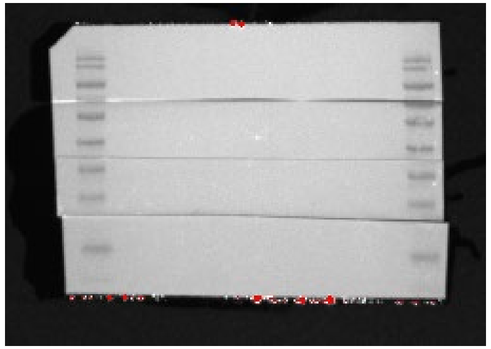

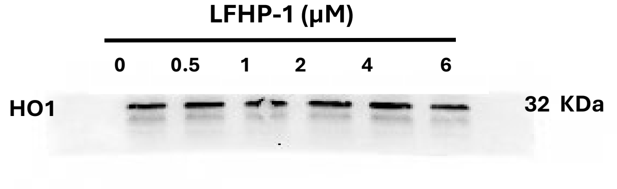

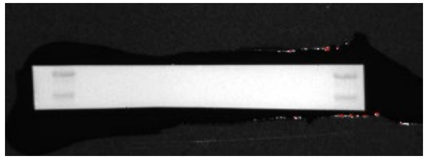

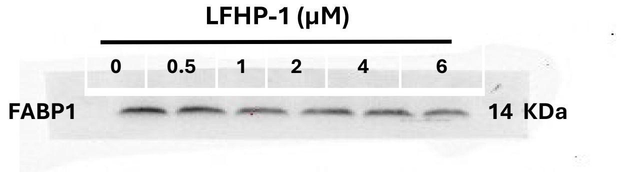

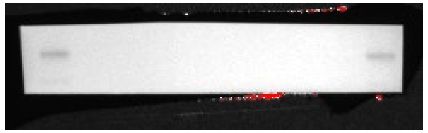

HUH7_LFHP-1_0.5-6 uM

EXPERIMENT 2

LFHP-1 (µM)

0 0.5 1 2 4 6

LIPIN 1

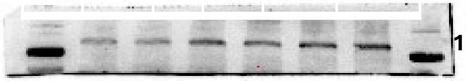

110 KDa

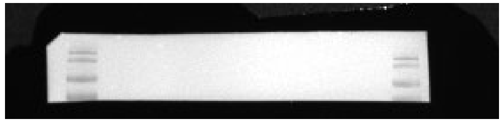

LFHP-1 (µM)

0 0.5 1 2 4 6

ACTIN

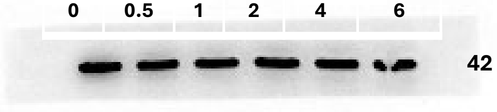

42 KDa

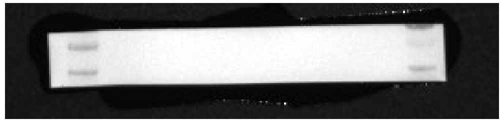

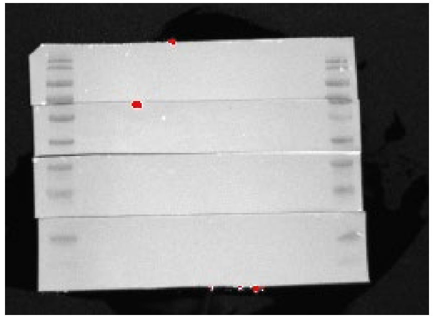

LFHP-1 (µM)

0 0.5 1 2 4 6

HO1

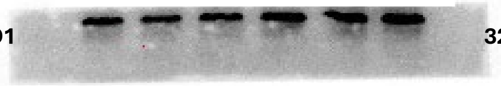

32 KDa

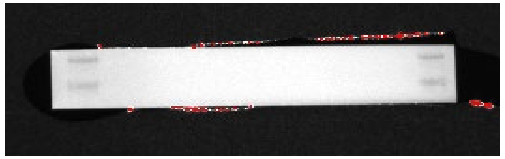

LFHP-1 (µM)

0 0.5 1 2 4 6

FABP1

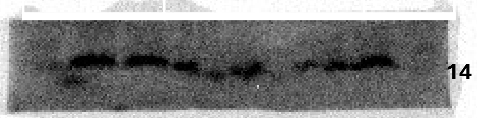

14 KDa

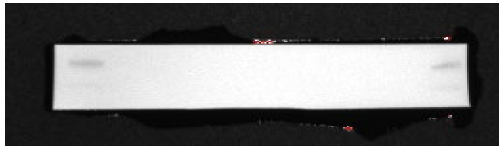

HEPG2_LFHP-1_0.5-6 uM

EXPERIMENT 3

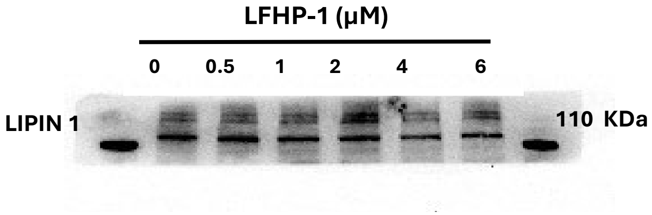

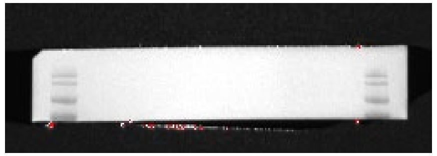

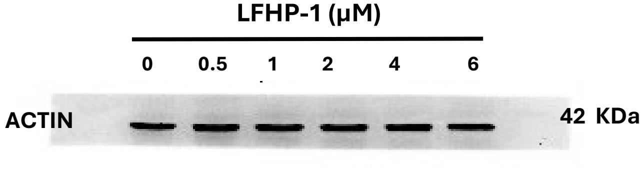

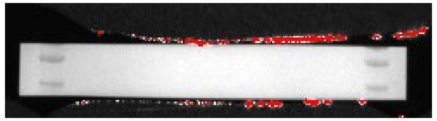

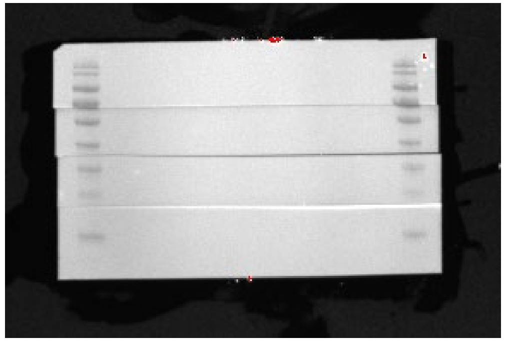

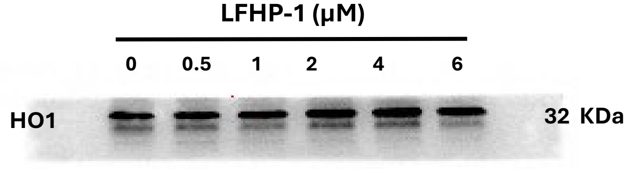

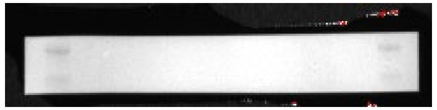

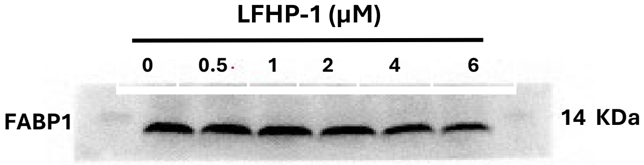

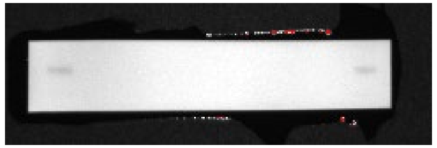

HUH7_LFHP-1_0.5-6 uM

EXPERIMENT 3

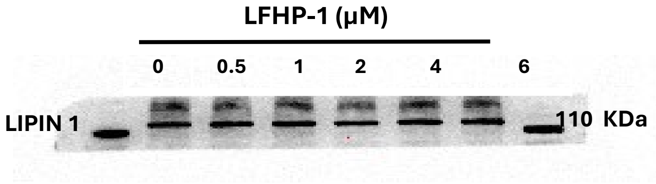

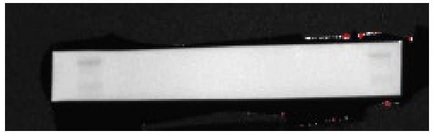

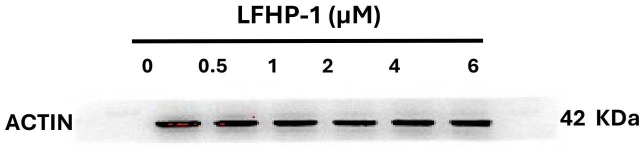

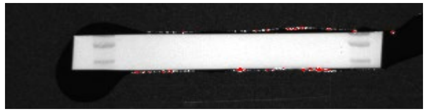

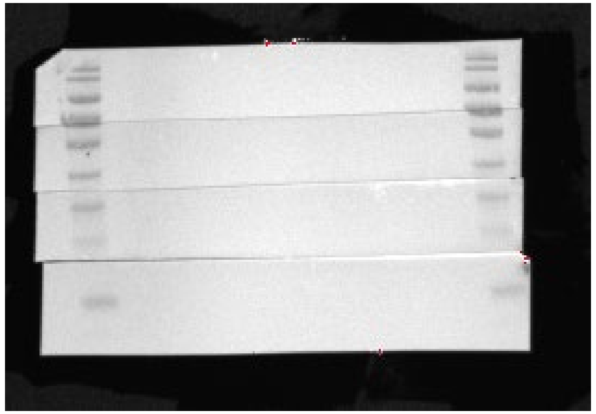

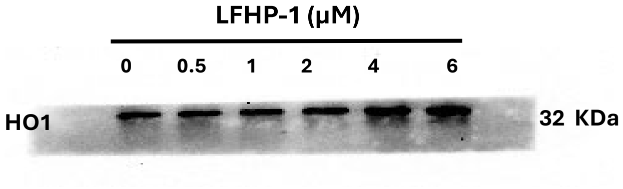

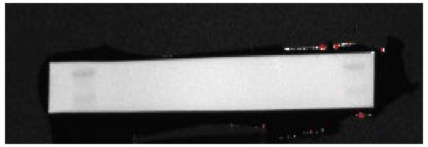

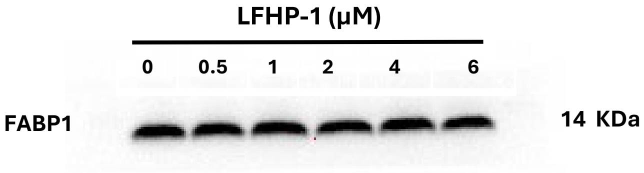

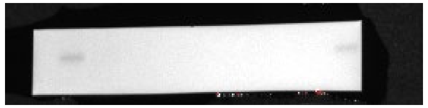

Immunoblot Densitometry Ratios

**Fig 2C**	*LFHP-1C*	FABP1			HO-1			Lipin-1			
**HepG2**	*Concentration*	Exp 1	Exp 2	Exp 3	Exp 1	Exp 2	Exp 3	Exp 1	Exp 2	Exp 3	
	0	1	1	1	1	1	1	1	1	1	
	0.5	0.894561	0.715146	0.893441	1.383348	1.052421	1.107705	0.85622	1.039175	0.98592	
	1	1.123739	0.823341	1.021437	1.673716	1.129583	1.23748	0.734907	1.48444	0.989098	
	2	1.534529	1.032517	0.956787	1.674971	1.377665	1.196686	0.980573	1.566263	1.031129	
	4	0.919444	0.680751	0.797505	1.75149	1.311253	1.70626	0.893336	1.015865	0.766797	
	6	0.855152	0.713431	0.64167	1.328657	1.320939	1.621382	0.891952	1.449725	0.714542	
**Fig 2D**	*LFHP-1C*	FABP1			HO-1			Lipin-1			
**Huh7**	*Concentration*	Exp 1	Exp 2	Exp 3	Exp 1	Exp 2	Exp 3	Exp 1	Exp 2	Exp 3	
	0	1	1	1	1	1	1	1	1	1	
	0.5	0.894561	0.715146	0.893441	1.383348	1.052421	1.107705	0.85622	1.039175	0.98592	
	1	1.123739	0.823341	1.021437	1.673716	1.129583	1.23748	0.734907	1.48444	0.989098	
	2	1.534529	1.032517	0.956787	1.674971	1.377665	1.196686	0.980573	1.566263	1.031129	
	4	0.919444	0.680751	0.797505	1.75149	1.311253	1.70626	0.893336	1.015865	0.766797	
	6	0.855152	0.713431	0.64167	1.328657	1.320939	1.621382	0.891952	1.449725	0.714542	
